# Analysis of the risk factors of radiation pneumonitis in patients after radiotherapy for esophageal squamous cell carcinoma

**DOI:** 10.3389/fonc.2023.1198872

**Published:** 2023-11-07

**Authors:** Lu Sun, Yan Wang, Lihua Zhu, Jun Chen, Zhifu Chen, Zhiyuan Qiu, Chaoyang Wu

**Affiliations:** ^1^ Department of Radiation Oncology, the People's Hospital Affiliated to Jiangsu University, Zhenjiang, Jiangsu, China; ^2^ Department of Oncology, the People’s Hospital Affiliated to Jiangsu University, Zhenjiang, Jiangsu, China

**Keywords:** esophageal squamous cell carcinoma (ESCC), radiation pneumonitis (RP), dose-volume-histogram, risk factors, V20

## Abstract

**Objective:**

To predict the risk factors of radiation pneumonitis (RP) in patients with esophageal squamous cell carcinoma (ESCC) who received radiotherapy.

**Methods:**

From January 2015 to October 2021, 477 ESCC patients were enrolled and were assessed retrospectively. All these patients received radiotherapy for primary lesions or mediastinal metastatic lymph nodes. Clinical efficacy and adverse events (AEs) were observed. Univariate analysis identified clinical and dosimetric factors associated with the development of RP, and multivariate logistic regression analysis identified independent potential risk factors associated with the development of RP. Nomograms were constructed to predict RP based on the results of multivariate logistic regression analysis.

**Results:**

Among the 477 ESCC patients, the incidence of RP was 22.2%, and the incidence of grade 4 or higher RP was 1.5%. Univariate analysis indicated that chronic obstructive pulmonary disease (COPD), pulmonary infection, leucopenia, PTV volume, V5, V20, V30 and MLD affected the occurrence of RP. The multivariate logistic regression analysis indicated that COPD (OR:1.821, 95%CI:1.111-2.985; *P*=0.017), pulmonary infection (OR:2.528, 95%CI:1.530-4.177; *P*<0.001), higher V20 (OR: 1.129, 95% CI:1.006-1.266; *P*=0.029) were significant independent predictors of RP in ESCC patients. COPD, pulmonary infection, V20 have been integrated for the RP nomogram. The rate of RP was significantly reduced in the V20<21.45% group. Further analysis indicated that the old age, diabetes, higher V20, and higher MLD were risk factors for grade 4 or higher RP. The area under the curve (AUC) value for V20 was 0.73 (95% CI, 0.567-0.893, *P* < 0.05).

**Conclusion:**

We have determined the risk factors of RP and grade 4 or higher RP in ESCC patients after radiotherapy. MLD, V20, COPD were independent factors for RP. It was necessary to take measures to reduce or avoid the occurrence of RP for patients with these risk factors at the early stage.

Esophageal carcinoma (EC) is one of the most common malignancies worldwide and there were nearly 604 thousand newly diagnosed cases of EC worldwide in 2020 (1). In China, the incidence and mortality of EC are higher (2), and according to the National Cancer Center, EC was the sixth most common cancer and the fifth most common cause of cancer-related deaths in 2016. Esophageal squamous cell carcinoma (ESCC) was the most common pathologic type, accounting for 90% of cases (3–5). Due to the lack of early symptoms and signs of EC, most patients have already lost the opportunity for surgery when diagnosed. Therefore, radiotherapy is one of the most important treatments for EC patients and has an irreplaceable position in the treatment of EC.

The esophagus has distinctive anatomical features that are closely related to the lungs. In the process of radiation for EC, lung tissue will inevitably be injured by radiation. The physiological characteristics of lung tissue are sensitive to radiation, therefore RP is one of the most common complications of radiation for EC (6). RP limits the radiation dose to the primary tumor and decreases the rate of local tumor control. High-grade RP severely affects the quality of life and long-term survival rate of patients. Continuous technological advances in radiotherapy have made it possible to apply radiation more precisely to the tumor while minimizing the dose to normal tissues, but the incidence of RP is still high (7, 8). The current treatment of RP mainly relies on symptomatic management such as glucocorticoids and antimicrobial drugs, which are poorly controlled in terms of overall efficacy (9). It is essential to predict the occurrence of RP at an early stage and to identify the relevant risk factors affecting the development of RP.

## Materials and methods

From January 2015 to October 2021, 477 ESCC patients were enrolled and were assessed retrospectively in the Affiliated People’s Hospital of Jiangsu University. All these patients received radiotherapy for primary lesions or mediastinal metastatic lymph nodes. Inclusion criteria were: (1) pathologically confirmed ESCC, (2) completed radiotherapy course and complete clinical records, (3) an Eastern Cooperative Oncology Group performance status (ECOG PS) of 0-2. Exclusion criteria were: (1) patients with contraindications to radiotherapy, (2) interruption of radiotherapy due to severe cardiopulmonary dysfunction (unrelated to radiotherapy), (3) patients had any other primary tumors or distant metastases. All patients were divided into two groups: the RP group and the No-RP group. The following indicators were recorded: gender, age, smoking, cT category, cN category, surgery, COPD, diabetes, occurrence of pulmonary infection during radiotherapy, leucopenia, hemoglobin, whether concurrent chemoradiotherapy was performed, radiation modality, whether reirradiation, PTV volume, radiotherapy dose, V5, V20, V30, MLD.

The radiotherapy equipment was Siemens Oncor and Medtronic Synergy VMAT linear accelerator, and the planning system was Pinnacle3. All patients received 3D-CRT or IMRT with involved field radiotherapy, and all the plans were implemented using linear accelerator and multileaf grating. The GTV, CTV and PTV were delineated according to the ICRU50 and ICRU62 reports. RT doses ranged from 41.4 to 64.8 Gy with a median dose of 54 Gy. Primary EC foci and mediastinal metastatic lymph nodes were placed externally 0.5 cm around and 2 cm above and below for CTV, and 0.5 cm outside of the CTV and 1 cm above and below for PTV. The delineation of organs at risk (OARs) based on the Radiotherapy and Oncology Group (RTOG) guidelines. The dose constraints were defined as follows: total lungs: V5 <70%, V20 <30%, V30 <18%, maximum point dose of the spinal cord <45 Gy; heart: V30 <40%, V40 <30%. All radiotherapy plans were certified according to standard requirements. The total prescribed dose of radiotherapy was determined on an individual patient basis at 1.8–2.0 Gy per fraction, once daily, and 5 fractions per week.

Adverse events (AEs) were evaluated according to National Cancer Institute Common Terminology Criteria for Adverse Events (NCI-CTCAE) version 4.0. The severity of radiation pneumonitis was graded according to the RTOG criteria.

### Statistical analysis

All statistical analyses were performed using SPSS (version 26.0; IBM Corporation, Armonk, NY, USA). Categorical variables were presented as numbers and percentages, and groups were compared using the χ2 test. Continuous variables were presented as means and standard deviations or percentiles, and groups were compared using the one-way ANOVA or the nonparametric Mann-Whitney U test. Logistic regression analysis was performed for those with significant single-factor analysis. A two-sided *P* < 0.05 was considered statistically significant. The nomogram applied to construct the scoring system was developed with independent risk factors based on multivariate logistic analysis using the rms package in R (version 3.6.0, R Development Core Team).

## Results

### Patient characteristics

**Figure 1:**
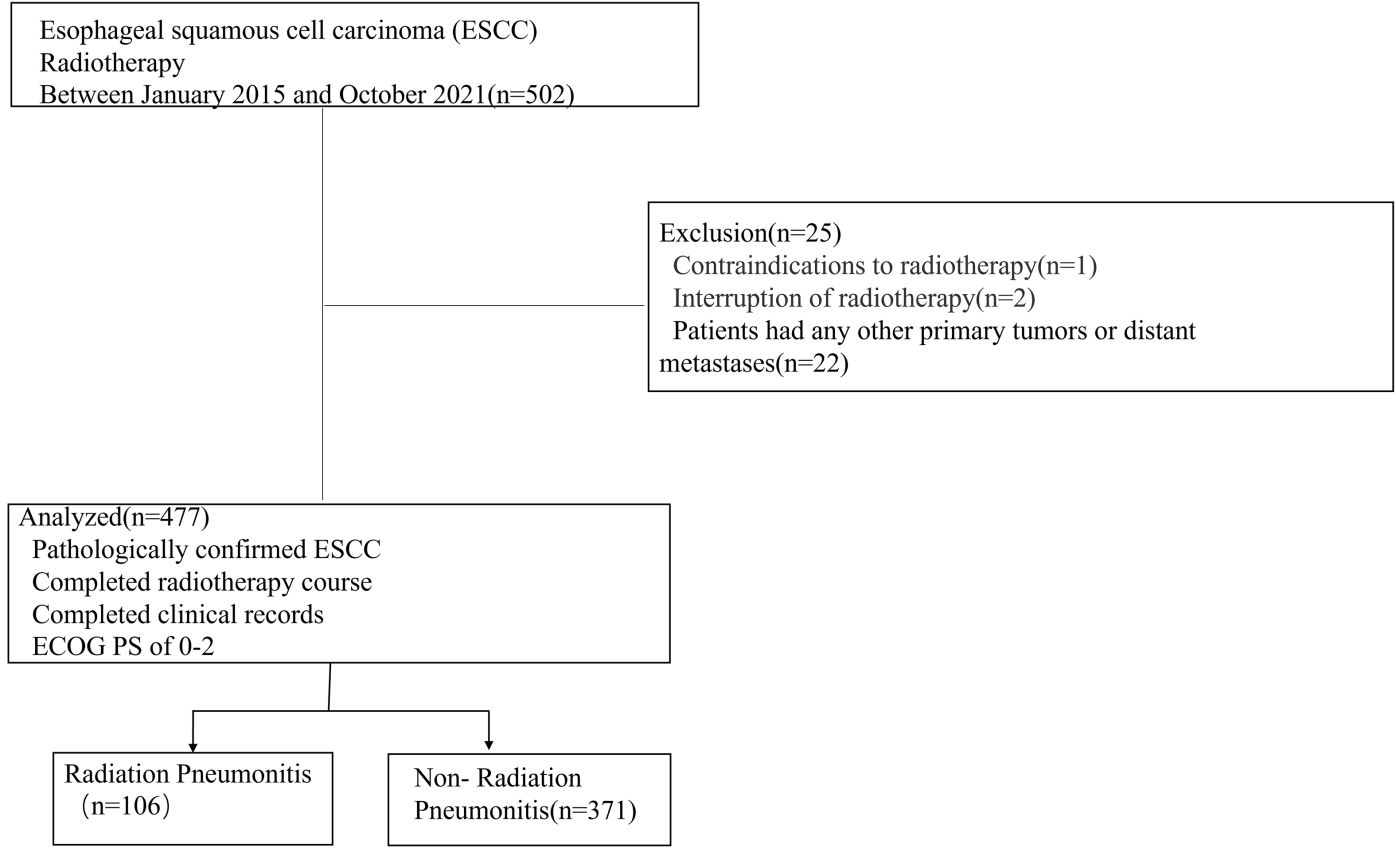
Flowchart of participant selection.

A total of 502 ESCC patients were enrolled in this study, including 353 men (74.0%) and 124 women (26.0%), with a median age of 68.4 ± 8.3 years (range: 32-88). Details of patient characteristics were shown in **Table 1**. Twenty-five patients were excluded according to the criteria. Finally, 477 patients were included. A detailed flowchart of patient selection was shown in **Figure 1**. 106 patients developed RP. 48 patients exhibited only grade 1 RP. The remaining were 9 patients with grade 2, 42 patients with grade 3, 4 patients with grade 4, and 3 patients with grade 5. Three patients died of RP. We found that 5 patients had RP during radiotherapy, 34 patients occurred within the first month after the end of radiotherapy, 36 patients occurred within the second month after the end of radiotherapy. In addition, there were 14 patients occurred RP in the third month after radiotherapy, and 17 patients occurred RP in the 3-6 months after the radiotherapy.

**Table 1:** Patient characteristics (n=477).

Variable	All patients n=477 (%)	RP-Group n=106(%)	No-RP Group n=371(%)	*P*-value
Gender				
Male	353(74.00)	81(76.42)	272(73.32)	0.521
Female	124(26.00)	25(23.58)	99(26.68)	
Age(years)				
≤65	151(31.66)	30(28.30)	121(32.61)	0.400
>65	326(68.34)	76(71.70)	250(67.39)	
Smoking				
No	292(61.22)	61(57.55)	231(62.26)	0.379
Yes	185(38.78)	45(42.45)	140(37.74)	
cT category				
1	51(10.69)	14(13.21)	37(9.97)	0.328
2	178(37.32)	40(37.74)	138(37.20)	
3	163(34.17)	39(36.79)	124(33.42)	
4	85(17.82)	13(12.26)	72(19.41)	
cN category				
cN0	156(32.70)	29(27.36)	127(34.23)	0.183
cN+	321(67.30)	77(72.64)	244(65.77)	
Surgery				
No	371(77.78)	88	283	0.664
Yes	106(22.22)	23	83	
COPD				
No	345(72.33)	64(60.38)	281(75.74)	0.002
Yes	132(27.67)	42(11.32)	90(24.26)	
Diabetes				
No	430(90.15)	94(88.68)	336(90.57)	0.565
Yes	47 (9.85)	12(11.32)	35(9.43)	
Pulmonary infection				
No	223(46.75)	30(28.30)	193(52.03)	0.000
Yes	254(53.25)	76(71.70)	178(47.98)	
Leucopenia				
0-2	302(63.31)	57 (53.77)	245(66.04)	0.021
3-4	175(36.69)	49(46.23)	126(33.96)	
Concurrent chemoradiotherapy				
No	202(42.35)	37(34.91)	165(44.47)	0.079
Yes	275(57.65)	69(65.09)	206(55.53)	
Radiation modality				
3D-CRT	88(18.45)	25(23.58)	63(16.98)	0.122
IMRT	389(81.55)	81(76.42)	308(83.02)	
Reirradiation				
No	428(89.73)	98(92.45)	330(88.95)	0.295
Yes	49(10.27)	8(7.55)	41(11.05)	
Hemoglobin Concentration at the beginning of RT(g/L)	117(106,127)	118.5(107,127.0)	117.0(105.0,127.0)	0.234
PTV volume(cm^3^)				
<160	40(8.39)	3(2.83)	37(9.97)	0.019
≥160	437(91.61)	103(97.17)	334(90.03)	
Radiotherapy dose (Gy)	5400(5050, 6120)	5400(5040,6120)	5400(5040,6020)	0.263
V5(%)	50.99(45.61, 54.52)	52.00(49.30,56.00)	50.65(45.00,54.14)	0.041
V20(%)	21.06(18.25, 22.64)	21.99(19.33,23.00)	21.00(17.82,22.53)	0.000
V30(%)	11.17(8.01,13.42)	12.06(9.57,14.00)	10.88(7.95,13.11)	0.000
MLD(Gy)	11.13(10.00,12.10)	11.36(10.71,12.39)	11.00(10.00,12.00)	0.007

### Univariate and multivariate analysis of RP after radiotherapy for ESCC

In the univariate analysis, COPD, pulmonary infection that occurring during RT, leucopenia, PTV volume, dosimetric factors (V5, V20, V30, and MLD) were associated with RP (*P*<0.05) (**Table 2**). Additionally, gender, age, smoking, concurrent chemotherapy, radiation modality, reirradiation, hemoglobin concentration at the beginning of RT, radiotherapy dose was not risk factors of RP (*P* > 0.05). Logistic regression analysis showed that COPD, the occurrence of pulmonary infection during RT, and elevated V20 were independent risk factors for RP in patients with ESCC (*P* < 0.05) (**Table 2**).

**Table 2:** Univariate and multivariate analysis for risk factors of RP in 477 ESCC patients.

Variable	RP-Group n=106(%)	No-RP Group n=371(%)	Univariate analysis, *P*-value	95%CI	Multivariate analysis, *P*-value	OR	95%CI
Gender							
Female Male	81(76.42) 25(23.58)	272(73.32) 99(26.68)	0.521	0.512-1.404			
Age(years)							
≤65 >65	30(28.30) 76(71.70)	121(32.61) 250(67.39)	0.400	0.762-1.972			
Smoking							
No Yes	61(57.55) 45(42.45)	231(62.26) 140(37.74)	0.380	0.785-1.887			
cT category							
1 2 3 4	14(13.21) 40(37.74) 39(36.79) 13(12.26)	37(9.97) 138(37.20) 124(33.42) 72(19.41)	0.155				
cN category							
cN0 cN+	29(27.36) 77(72.64)	127(34.23) 244(65.77)	0.185	0.857-2.229			
Surgery			0.664	0.530-1.499			
	88 23	283 83					
COPD							
No Yes	64(60.38) 42(11.32)	281(75.74) 90(24.26)	0.002	1.299-3.232	0.017	1.821	1.111-2.985
Diabetes							
No Yes	94(88.68) 12(11.32)	336(90.57) 35(9.43)	0.566	0.612-2.454			
Pulmonary infection							
No Yes	30(28.30) 76(71.70)	193(52.03) 178(47.98)	0.000	1.718-4.391	0.000	2.528	1.530-4.177
Leucopenia							
0-2 3-4	57 (53.77) 49(46.23)	245(66.04) 126(33.96)	0.022	1.079-2.591	0.237	1.326	0.831-2.117
Concurrent chemoradiotherapy							
No Yes	37(34.91) 69(65.09)	165(44.47) 206(55.53)	0.080	0.954-2.340			
Radiation modality							
3D-CRT IMRT	25(23.58) 81(76.42)	63(16.98) 308(83.02)	0.124	0.392-1.119			
Reirradiation							
No Yes	98(92.45) 8(7.55)	330(88.95) 41(11.05)	0.298	0.298-1.448			
Hemoglobin Concentration at the beginning of RT(g/L)	118.5(107,127.0)	117.0(105.0,127.0)	0.355	0.993-1.019			
PTV volume(cm^3^)							
<160 ≥160	3(2.83) 103(97.17)	37(9.97) 334(90.03)	0.029	1.149-12.591	0.351	1.847	0.509-6.698
Radiotherapy dose(Gy)	5400(5040,6120)	5400(5040,6020)	0.305	1.000-1.001			
V5(%)	52.00(49.30,56.00)	50.65(45.00,54.14)	0.012	1.006-1.049	0.910	1.003	0.959-1.048
V20(%)	21.99(19.33,23.00)	21.00(17.82,22.53)	0.000	1.063-1.199	0.039	1.129	1.006-1.266
V30(%)	12.06(9.57,14.00)	10.88(7.95,13.11)	0.009	1.019-1.141	0.744	1.013	0.937-1.095
MLD(Gy)	11.36(10.71,12.39)	11.00(10.00,12.00)	0.001	1.069-1.322	0.881	0.980	0.749-1.282

**Figure 2:**
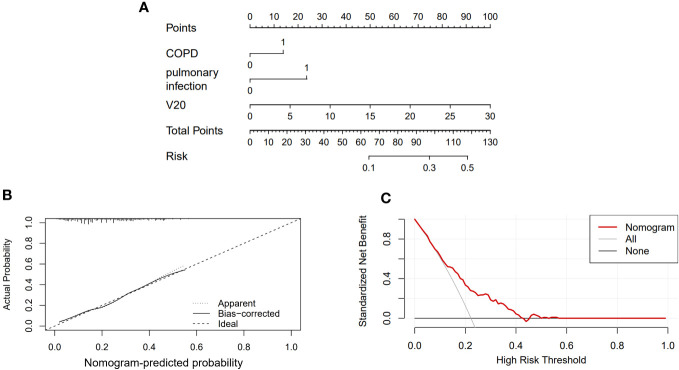
Nomogram of the probability of RP in the ESCC patients. **(A)** Nomogram of the probability of RP. **(B)** Calibration curve of the nomogram. **(C)** DCA of the nomogram.

A nomogram was constructed to predict the risk of RP in ESCC patients treated with radiotherapy. This model included three predictors: (**Figure 2A**): COPD (Yes=1 or N=0), the occurrence of pulmonary infection during RT (Yes=1 or No=0) and V20. For example, a patient with COPD, pulmonary infection, V20 (25%) was given a total of 110 points for RP (13 points for COPD, 24 points for pulmonary infection, 83 points for V20). This indicated that the risk of RP was over 40% in this patient. The calibration curve showed that this diagnostic nomogram had a good calibration (**Figure 2B**). Moreover, decision curve analysis (DCA) was applied to evaluate the clinical utility of the diagnostic nomogram, as shown in **Figure 2C**.

### The optimal cutoff values for dosimetry parameters

The optimal cutoff values for the dosimetry parameters were confirmed using a receiver operating characteristic (ROC) curve. As shown in **Table 3**. Controlling V5 within 51.47%, V20 within 21.45%, V30 within 10.54%, and MLD within 10.98% could protect patients from RP and reduced the probability of RP significantly.

**Table 3:** The optimal cutoff value for dosimetric parameter to protect from RP.

Variable	RP Group	No- RP Group	*P*-Value
V5			
<51.47	44	214	0.003
≥51.47	62	157	
V20			
<21.45	44	218	0.002
≥21.45	62	153	
V30			
<10.54	32	175	0.002
≥10.54	74	196	
MLD			
<10.98	30	161	0.005
≥10.98	76	210	

### Univariate analysis for risk factors of grade-4 or higher RP

A portion of patients developed grade 4 or higher RP (7 of 106), which was life-threatening or even fatal. In this study, three patients died of RP. To reduce the risk of patients developing grade 4 or higher RP and to find common risk factors affecting these patients, we reanalyzed 106 patients with RP, which were shown in **Table 4**.

**Table 4:** Univariate analysis for risk factors of grade 4 or higher RP.

Various	Grade 1-3 RP	Grade 4-5 RP	*P*-Value
Gender			
Male	74	7	0.195
Female	25	0	
Age	68.69 ± 7.37	75.00 ± 7.59	0.034
Smoking			
No	58	3	0.454
Yes	41	4	
COPD			
No	62	2	0.111
Yes	37	5	
Diabetes			
No	90	4	0.035
Yes	9	3	
Pulmonary infection			
No	28	2	1.000
Yes	71	5	
Leucopenia stage			
0-2	52	5	0.447
3-4	47	2	
Concurrent chemoradiotherapy			
No	34	3	0.693
Yes	65	4	
Radiation modality			
3D-CRT	22	3	0.434
IMRT	77	4	
Reirradiation			
No	91	7	0.967
Yes	8	0	
Concentration at the beginning of RT(g/L)	117.25 ± 1.57	121.29 ± 4.59	0.506
PTV volume(cm^3^)	327.21(244.48,409.59)	369.00(244.00,663.22)	0.192
Radiotherapy dose(Gy)	5400(5040,6120)	5940(5040,6300)	0.367
V5(%)	52.00(47.00,56.00)	52.00(49.00,62.00)	0.670
V20(%)	21.88(19.83,23.00)	23.00 (22.00,27.00)	0.042
V30(%)	11.82 ± 3.00	14.15 ± 3.33	0.052
MLD(Gy)	11.47 ± 1.43	12.81 ± 1.79	0.019

**Figure 3:**
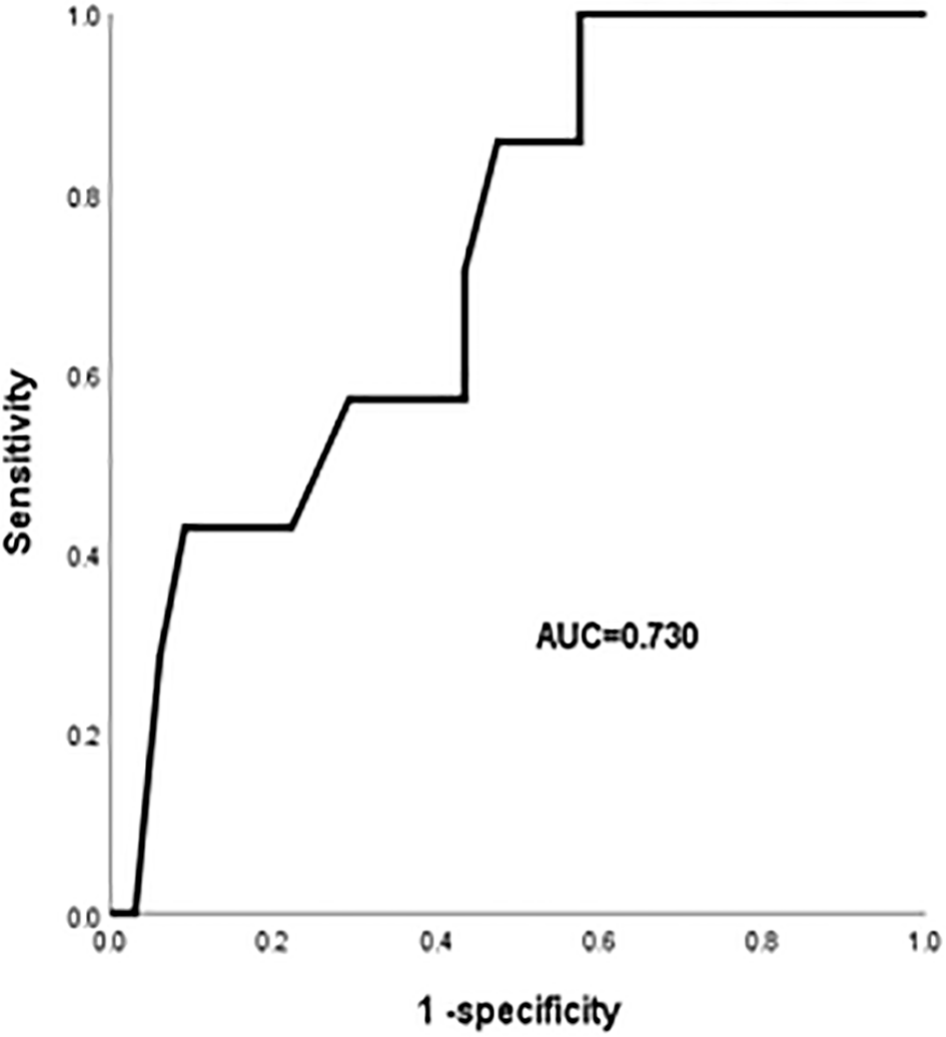
ROC curve of V20 in predicting the occurrence of grade 4 or higher RP.

Univariate analysis showed that age, diabetes, high V20 and MLD were significantly associated with the occurrence of grade 4 or higher RP (*P*< 0.05). The ROC curve was performed to analyze age, diabetes, V20, and MLD levels separately for predicting RP and to determine the area under the curve (AUC) and the optimal cut-off value. The results showed that V20 was the best predictor (*P*<0.05) with an AUC of 0.73 (95%CI:0.567-0.893), an optimal cut-off value of 21.18%. **Figure 3** showed the ROC curve of V20 for predicting the occurrence of grade 4 or higher RP.

## Discussion

EC is a common gastrointestinal cancer, and squamous cell carcinoma and adenocarcinoma are the main pathologic subtypes of EC. In China, predominant histological type of EC is squamous cell carcinoma (approximately 90%) (4, 5). Radiotherapy plays an essential role in cancer treatment, especially in ESCC, which is highly sensitive to radiation. Radiotherapy increases the survival rate and improves the prognosis of EC patients by reducing the risk of distant metastasis and relapse. The higher radiation doses have been associated with the superior local control rate of ESCC (10). However, radiotherapy can cause various AEs, and RP is one of the most common AEs in EC. RP is a major factor limiting the dose of radiotherapy, which not only influences the completion of radiotherapy but also decreases the survival benefit of the patient, especially for grade 4-5 RP. The aim of the present study was to determine the risk factors associated with the occurrence of RP and grade 4 or higher RP in ESCC patient receiving radiotherapy. Our study showed that the incidence of RP in ESCC patients was 22.2% and the incidence of grade 4 and grade 5 RP was 1.5%, which is similar to the previous reports (11, 12).

In recent years, clinical practitioners have tried to find the most applicable risk factors for predicting RP, such as age (13–15), smoking (16, 17), and relevant dosimetry parameters in RT (18–20), but the results were still contradictory. In the present study, there were no statistically significant differences between smoking and the occurrence of RP. Vogelius et al. reported a lower incidence of RP in smokers compared with nonsmokers (21), whereas Monson JM et al. reported a higher incidence of RP in smokers (22). Whether smoking is a risk factor for RP remains a topic of debate. Gender was not considered a risk factor for inducing RP in our study, which is consistent with previous findings (21). We found that there were no statistically significant differences between age and RP. However, Jin H et al. reported that older patients may have comorbidities and reduced lung function, which may increase the risk of RP (16). Univariate and multifactorial analyses revealed that COPD, pulmonary infection during RT and higher V20 were significantly associated with the development of RP and were independent risk factors predicting the development of RP.

The predictive value of COPD is controversial, which was consistent previous publications (23, 24). Studies about the occurrence of RP in EC patients combined COPD are limited. Some studies about lung cancer have shown that patients combined COPD had an increased risk of RP (24, 25), but other reports held the opposite view that the risk of RP was not relevant to COPD (16, 26). Our study indicated that patients with COPD had a higher risk of suffering from RP, while COPD was not associated with grade 4 or higher RP. Patients were more susceptible to RP if they developed a pulmonary infection during RT. The mechanism is that lung infections cause an increase in inflammatory cytokines, and chronic inflammation damages lung tissue. Injured lung tissue suffered from increased sensitivity to radiation and weakened self-healing capacity (27). Therefore, it is important to pay special attention and provide early intervention for patients with ESCC who have these risk factors.

Leucopenia is a common AE of chemoradiotherapy. Univariate analysis indicated that the rate of RP was 38.9% in the group of grades 3-4 leucopenia, which was higher than the other group of leucopenia stage below grade 3. The susceptibility to RP was associated with the severity of leucopenia. The mechanism is that radiotherapy provokes myelosuppression and decreases leucocyte, which results in immunosuppression and susceptibility to infection (28).

Dosimetry parameters were considered to play a crucial role in the development of RP. It was an effective measure to reduce the incidence of RP by controlling dosimetry parameters strictly. In the present study, PTV-volume, V5, V20, V30, MLD were associated with RP, but multivariate analysis indicated that only V20 was independent influencing factor of RP. However, some researchers suggested that V30 may be the better predictor of the occurrence of RP based on lung cancer (29, 30). Therefore, more data are needed to validate the dosimetry parameters that predict the incidence of RP in ESCC. For further clinical guidance and optimization of dosimetry parameters, we used ROC curves to determine the optimal limiting range of relevant dosimetry parameters. The study showed that the probability of RP was significantly lower in those with V20 <21.45% compared with those with V20 ≥21.45% (20.18% vs 40.52%, *P*=0.002). In clinical practice, it is very important to reduce the risk of RP by limiting V20 to less than 21.5%.

In recent years, with the development of analytical methods, the construction of mathematical models based on multi-index has been increasingly applied in the field of medicine (31–33). This approach combines a number of important parameters to generate a predictive model to achieve a better diagnostic performance. In the present study, we selected the most significant indices based on the multivariate analysis to construct a predictive model. In the practical application of the nomogram, we only need to convert the corresponding predictor value into the corresponding nomogram score value, and then add the score values to obtain the total score. Then the risk incidence corresponding to the total score is obtained, as described in the results section. The operation of the nomogram is simple and intuitive, without complicated calculation, less time-consuming, easy to use and can be popularized quickly.

In our study, the incidence of RP above grade 4 was 1.5%, and older age, diabetes, high V20 and MLD could cause grade 4 or higher RP. Although there was no statistical difference in the occurrence of RP in combination with diabetes, our data suggest that the occurrence of RP in patients with diabetes leads to the appearance of RP above grade 4. In the hyperglycemic environment of diabetic patients, changes such as fibrin-like degeneration and fat necrosis in the alveolar basement membrane cause increased permeability of the vascular wall and aggravate extravasation at sites where inflammation occurs (34). In addition, chronic hyperglycemia could lead to the imbalance of lymphocyte, impaired cellular immunity, result in susceptibility to various pulmonary infections and aggravate the symptoms of RP (35). If the ESCC patients had diabetes, the level of V20 and MLD should be strictly controlled in order to avoid the occurrence of grade 2 or higher RP and to alleviate the clinical symptoms of RP.

Few controlled studies have been conducted to evaluate the role of various therapies in RP patients. For mild symptoms, clinical observation can be considered. Glucocorticoids reduce inflammation and inhibit lymphocyte and endothelial cell toxicity, and systemic glucocorticoids to treat significantly symptomatic RP; a dose of 60-100 mg/day of prednisone for 2 weeks followed by a slow taper over 3-12 weeks (36). Supportive care with antibiotics, oxygen and anti-tussive therapy is also helpful. However, recent advances in molecular mechanisms of RP have led to identification of several potential targets for therapy.

In this study, we focused on the risk factors associated with the development of RP in ESCC patients. With a detailed classification of RP, we investigated the risk factors associated with grade 4 or higher RP for the first time. However, there were still some limitations: (1) the study was retrospective; (2) the number of samples is small; (3) only ESCC were included in this study, and esophageal adenocarcinoma needs further investigation.

In conclusion, our study confirmed the risk factors for the occurrence of RP and RP above grade 4 for ESCC patients in RT. Dosimetric parameters such as V20 and clinical features such as COPD are closely related to the occurrence of RP. For patients with these risk factors, taking effective measures at early stage will reduce and prevent the occurrence of RP. Next, we will explore the risk factors associated with radiation-induced pulmonary fibrosis in order to improve the quality of patient survival.

## Data availability statement

The raw data supporting the conclusions of this article will be made available by the authors, without undue reservation.

## Ethics statement

The studies involving humans were approved by Ethics Committee of People’s Hospital affiliated to Jiangsu University. The studies were conducted in accordance with the local legislation and institutional requirements. Written informed consent for participation was not required from the participants or the participants’ legal guardians/next of kin in accordance with the national legislation and institutional requirements.

## Author contributions

Study concepts: LS, YW, CW, ZQ, LZ, JC, ZC. Study design: LS, YW,CW, ZQ, LZ, JC, ZC. Data acquisition: LS, YW, ZC. Quality control of data and algorithms: CW, ZQ, JC. Data analysis and interpretation: LS, YW. Statistical analysis: LS, YW, LZ. Manuscript preparation: LS, YW, CW. Manuscript editing: LS, YW. Manuscript review: CW, ZQ. All authors contributed to the article and approved the submitted version.

## References

[1] Sung H Ferlay J Siegel RL Laversanne M Soerjomataram I Jemal A. Global cancer statistics 2020: GLOBOCAN estimates of incidence and mortality worldwide for 36 cancers in 185 countries. CA Cancer J Clin (2021) 71(3):209–49. doi: 10.3322/caac.21660 33538338

[2] Chen W Zheng R Baade PD Zhang S Zeng H Bray F. Cancer statistics in China, 2015. CA Cancer J Clin (2016) 66(2):115–32. doi: 10.3322/caac.21338 26808342

[3] Zheng R Zhang S Zeng H Wang S Sun K Chen R. Cancer incidence and mortality in China, 2016. J Natl Cancer Center (2022) 2(1):1–9. doi: 10.1016/j.jncc.2022.02.002 PMC11256658 39035212

[4] Pennathur A Gibson MK Jobe BA Luketich JD. Oesophageal carcinoma. Lancet (2013) 381(9864):400–12. doi: 10.1016/S0140-6736(12)60643-6 23374478

[5] Jemal A Bray F Center MM Ferlay J Ward E Forman D. Global cancer statistics. CA Cancer J Clin (2011) 61(2):69–90. doi: 10.3322/caac.20107 21296855

[6] Ullah T Patel H Pena GM Shah R Fein AM. A contemporary review of radiation pneumonitis. Curr Opin Pulm Med (2020) 26(4):321–5. doi: 10.1097/MCP.0000000000000682 32427626

[7] Robbins ME Brunso-Bechtold JK Peiffer AM Tsien CI Bailey JE Marks LB. Imaging radiation-induced normal tissue injury. Radiat Res (2012) 177(4):449–66. doi: 10.1667/RR2530.1 PMC3733443 22348250

[8] Koenig TR Munden RF Erasmus JJ Sabloff BS Gladish GW Komaki R. Radiation injury of the lung after three-dimensional conformal radiation therapy. AJR Am J Roentgenol (2002) 178(6):1383–8. doi: 10.2214/ajr.178.6.1781383 12034601

[9] Bledsoe TJ Nath SK Decker RH. Radiation pneumonitis. Clin Chest Med (2017) 38(2):201–8. doi: 10.1016/j.ccm.2016.12.004 28477633

[10] Mcdowell LJ Huang SH Xu W Che J Wong RKS Brierley J. Effect of intensity modulated radiation therapy with concurrent chemotherapy on survival for patients with cervical esophageal carcinoma. Int J Radiat Oncol Biol Phys (2017) 98(1):186–95. doi: 10.1016/j.ijrobp.2017.01.003 28258892

[11] Zhao Y Chen L Zhang S Wu Q Jiang X Zhu H. Predictive factors for acute radiation pneumonitis in postoperative intensity modulated radiation therapy and volumetric modulated arc therapy of esophageal cancer. Thorac Cancer (2015) 6(1):49–57. doi: 10.1111/1759-7714.12142 26273335 PMC4448459

[12] Shaikh T Churilla TM Monpara P Scott WJ Cohen SJ Meyer JE. Risk of radiation pneumonitis in patients receiving taxane-based trimodality therapy for locally advanced esophageal cancer. Pract Radiat Oncol (2016) 6(6):388–94. doi: 10.1016/j.prro.2016.02.004 PMC5002262 27025161

[13] Parashar B Edwards A Mehta R Pasmantier M Wernicke AG Sabbas A. Chemotherapy significantly increases the risk of radiation pneumonitis in radiation therapy of advanced lung cancer. Am J Clin Oncol (2011) 34(2):160–4. doi: 10.1097/COC.0b013e3181d6b40f 20498591

[14] Palma DA Senan S Tsujino K Barriger RB Rengan R Moreno M. Predicting radiation pneumonitis after chemoradiation therapy for lung cancer: an international individual patient data meta-analysis. Int J Radiat Oncol Biol Phys (2013) 85(2):444–50. doi: 10.1016/j.ijrobp.2012.04.043 PMC3448004 22682812

[15] Tsujino K Hashimoto T Shimada T Yoden E Fujii O Ota Y. Combined analysis of V20, VS5, pulmonary fibrosis score on baseline computed tomography, and patient age improves prediction of severe radiation pneumonitis after concurrent chemoradiotherapy for locally advanced non-small-cell lung cancer. J Thorac Oncol (2014) 9(7):983–90. doi: 10.1097/JTO.0000000000000187 24922010

[16] Jin H Tucker SL Liu HH Wei X Yom SS Wang S. Dose-volume thresholds and smoking status for the risk of treatment-related pneumonitis in inoperable non-small cell lung cancer treated with definitive radiotherapy. Radiother Oncol (2009) 91(3):427–32. doi: 10.1016/j.radonc.2008.09.009 PMC5555233 18937989

[17] Mörth C Kafantaris I Castegren M Valachis A. Validation and optimization of a predictive model for radiation pneumonitis in patients with lung cancer. Oncol Lett (2016) 12(2):1144–8. doi: 10.3892/ol.2016.4678 PMC4950220 27446409

[18] Bradley JD Moughan J Graham MV Byhardt R Govindan R Fowler J. A phase I/II radiation dose escalation study with concurrent chemotherapy for patients with inoperable stages I to III non-small-cell lung cancer: phase I results of RTOG 0117. Int J Radiat Oncol Biol Phys (2010) 77(2):367–72. doi: 10.1016/j.ijrobp.2009.04.029 PMC2869096 20457350

[19] Asakura H Hashimoto T Zenda S Harada H Hirakawa K Mizumoto M. Analysis of dose-volume histogram parameters for radiation pneumonitis after definitive concurrent chemoradiotherapy for esophageal cancer. Radiother Oncol (2010) 95(2):240–4. doi: 10.1016/j.radonc.2010.02.006 20223539

[20] Nomura M Kodaira T Furutani K Tachibana H Tomita N Goto Y. Predictive factors for radiation pneumonitis in oesophageal cancer patients treated with chemoradiotherapy without prophylactic nodal irradiation. Br J Radiol (2012) 85(1014):813–8. doi: 10.1259/bjr/13604628 PMC3474116 22253344

[21] Kong F-MS Wang S. Nondosimetric risk factors for radiation-induced lung toxicity. Semin Radiat Oncol (2015) 25(2):100–9. doi: 10.1016/j.semradonc.2014.12.003 PMC4400801 25771414

[22] Monson JM Stark P Reilly JJ Sugarbaker DJ Strauss GM Swanson SJ. Clinical radiation pneumonitis and radiographic changes after thoracic radiation therapy for lung carcinoma. Cancer (1998) 82(5):842–50. doi: 10.1002/(SICI)1097-0142(19980301)82:5<842::AID-CNCR7>3.0.CO;2-L 9486572

[23] Inoue T Shiomi H Oh R-J. Stereotactic body radiotherapy for Stage I lung cancer with chronic obstructive pulmonary disease: special reference to survival and radiation-induced pneumonitis. J Radiat Res (2015) 56(4):727–34. doi: 10.1093/jrr/rrv019 PMC4497392 25887042

[24] Kimura T Togami T Takashima H Nishiyama Y Ohkawa M Nagata Y. Radiation pneumonitis in patients with lung and mediastinal tumours: a retrospective study of risk factors focused on pulmonary emphysema. Br J Radiol (2012) 85(1010):135–41. doi: 10.1259/bjr/32629867 PMC3473945 21385918

[25] Rancati T Ceresoli GL Gagliardi G Schipani S Cattaneo GM. Factors predicting radiation pneumonitis in lung cancer patients: a retrospective study. Radiother Oncol (2003) 67(3):275–83. doi: 10.1016/S0167-8140(03)00119-1 12865175

[26] Takeda A Kunieda E Ohashi T Aoki Y Oku Y Enomoto T. Severe COPD is correlated with mild radiation pneumonitis following stereotactic body radiotherapy. Chest (2012) 141(4):858–66. doi: 10.1378/chest.11-1193 21885726

[27] Cousin F Desir C Ben Mustapha S Mievis C Coucke P Hustinx R. Incidence, risk factors, and CT characteristics of radiation recall pneumonitis induced by immune checkpoint inhibitor in lung cancer. Radiother Oncol (2021) 157:47–55. doi: 10.1016/j.radonc.2021.01.001 33453313

[28] Telarovic I Yong CSM Guckenberger M Unkelbach J Pruschy M. Radiation-induced lymphopenia does not impact treatment efficacy in a mouse tumor model. Neoplasia (2022) 31:100812. doi: 10.1016/j.neo.2022.100812 35667149 PMC9168138

[29] Kim M Lee J Ha B Lee R Lee K-J Suh HS. Factors predicting radiation pneumonitis in locally advanced non-small cell lung cancer. Radiat Oncol J (2011) 29(3):181–90. doi: 10.3857/roj.2011.29.3.181 PMC3429901 22984669

[30] Roeder F Friedrich J Timke C Kappes J Huber P Krempien R. Correlation of patient-related factors and dose-volume histogram parameters with the onset of radiation pneumonitis in patients with small cell lung cancer. Strahlenther Onkol (2010) 186(3):149–56. doi: 10.1007/s00066-010-2018-4 20165822

[31] Liu A Wang Z Yang Y Wang J Dai X Wang L. Preoperative diagnosis of Malignant pulmonary nodules in lung cancer screening with a radiomics nomogram. Cancer Commun (Lond) (2020) 40(1):16–24. doi: 10.1002/cac2.12002 32125097 PMC7163925

[32] Chen D Fu M Chi L Lin L Cheng J Xue W. Prognostic and predictive value of a pathomics signature in gastric cancer. Nat Commun (2022) 13(1):6903. doi: 10.1038/s41467-022-34703-w 36371443 PMC9653436

[33] Liu J Huang X Yang W Li C Li Z Zhang C. Nomogram for predicting overall survival in stage II-III colorectal cancer. Cancer Med (2020) 9(7):2363–71. doi: 10.1002/cam4.2896 PMC7131840 32027098

[34] Malenica M Šilar M Dujić T Bego T Semiz S Škrbo S. Importance of inflammatory markers and IL-6 for diagnosis and follow up of patients with type 2 diabetes mellitus. Med Glas (Zenica) (2017) 14(2):169–75. doi: 10.17392/920-17 28786970

[35] Wu H-P Chu C-M Lin C-Y Yu C-C Hua C-C Yu T-J. Liver cirrhosis and diabetes mellitus are risk factors for Staphylococcus aureus infection in patients with healthcare-associated or hospital-acquired pneumonia. Pulm Med (2016) 2016:4706150. doi: 10.1155/2016/4706150 26998356 PMC4779838

[36] Graves PR Siddiqui F Anscher MS Movsas B. Radiation pulmonary toxicity: from mechanisms to management. Semin Radiat Oncol (2010) 20(3):201–7. doi: 10.1016/j.semradonc.2010.01.010 20685583

